# Effect of the location and size of thyroid nodules on the diagnostic performance of ultrasound elastography: A retrospective analysis

**DOI:** 10.6061/clinics/2020/e1720

**Published:** 2020-06-16

**Authors:** Xinxin Xie, Yongqiang Yu

**Affiliations:** I Department of Ultrasound, The First Affiliated Hospital of Anhui Medical University, Hefei, Anhui, China, 230022; II Department of Radiology, The First Affiliated Hospital of Anhui Medical University, Hefei, Anhui, China, 230022

**Keywords:** Benign Nodules, Fine-Needle Aspiration Cytopathology, Grayscale Ultrasound, Suspicious Nodules, Ultrasound Elastography, Thyroid Nodules

## Abstract

**OBJECTIVES:**

Ultrasound-guided fine-needle aspiration biopsies are recommended for the detection of suspicious thyroid nodules. However, the best approach regarding suspicious ultrasound features for thyroid nodules is still unclear. This study aimed to evaluate the effect of location and size of thyroid nodules on the diagnostic performance of strain ultrasound elastography. In addition, this study evaluated whether ultrasound elastography predicts malignancy in thyroid nodules.

**METHODS:**

Data regarding the size, depth, and distance from the carotid artery of nodules, the elasticity contrast index, and the nature of nodules were analyzed.

**RESULTS:**

There was no significant difference in the depth (*p*=0.092) and the distance from the carotid artery (*p*=0.061) between benign and suspicious nodules. Suspicious nodules were smaller than benign nodules (*p*<0.0001, *q*=23.84) and had a higher elasticity contrast index (*p*<0.0001, *q*=21.05). The depth of nodules and the size of the nodule were not associated with the correct value of the elasticity contrast index (*p*>0.05 for both). The diagnostic performance of ultrasound elastography was not affected by the distance of the nodules from the carotid artery if they were located ≥15 mm from the carotid artery (*p*=0.5960). However, if the suspicious nodules were located <15 mm from the carotid artery, the diagnostic accuracy was hampered (*p*=0.006).

**CONCLUSIONS:**

The strain ultrasound elastography should be carefully evaluated when small thyroid nodules are located near the carotid artery.

## INTRODUCTION

Thyroid nodules that present without symptoms are common in clinical practice (1). Most thyroid nodules are benign (2). Computed tomography and magnetic resonance imaging are not successful in the differentiation of benign and suspicious nodules (2,3). Therefore, ultrasound-guided fine-needle aspiration cytology is recommended in the management of thyroid nodules (4). Grayscale ultrasound is a sensitive method for the diagnosis of known features of the thyroid gland like solidity, echogenicity, calcification, and lobulation (5). Despite the high sensitivity and low specificity rates in B-mode ultrasound findings, there are several guidelines to increase specificity rates of suspicious nodules in B-mode ultrasound findings in different criteria, for example, the 2015 American Thyroid Association (ATA) management guidelines suggest a pattern-based approach for risk stratification and give the estimated risks of malignancy for each category (6). However, they suggest more conservative approaches than the other guidelines regarding the diagnosis of differentiated thyroid cancers (7). The Korean Thyroid Association (KTA)/Korean Society of Thyroid Radiology (KSThR) proposed a simpler pattern-based approach in 2016. These features are a combination of solidity, echogenicity, and suspicious ultrasound features, and can be easily substituted for the ATA guidelines (8) but have a different sensitivity and accuracy than the ATA guidelines (9). The American College of Radiology Thyroid Imaging, Reporting and Data System (ACR TI-RADS) classification published in 2017 assigns points for all ultrasound features of a nodule (10), but it is rather complex (11). The clinical efficacy or the complementary role of elastography in the diagnosis of thyroid nodules is controversial (12). Therefore, the best approach for suspicious ultrasound features for thyroid nodules is still unclear (13).

In addition, the efficacy of ultrasound can depend on the skill and experience of the ultrasound technologist (14). Ultrasound elastography is based on the stiffness of tissues and improves the sensitivity of grayscale ultrasound (15) but has not improved the accuracy and specificity of grayscale ultrasound (16). Strain elastography, and in particular, external compression elastography is a method now known to have many limitations, including interference from carotid artery pulsations and poor characterization of large nodules (2). Texture analysis of grayscale ultrasound is generally performed for the discrimination of the thyroid nodules (17).

This retrospective study aimed to evaluate the effect of location and size of thyroid nodules on the diagnostic performance of strain ultrasound elastography. In addition, this study evaluates whether ultrasound elastography can predict malignancy in thyroid nodules considering the results of fine-needle aspiration cytopathology as the reference standard.

## MATERIALS AND METHODS

### Ethics approval and consent to participate

The designed protocol (AFF1/CL/18/19 dated 13 November 2019) of the established study was approved by the First Affiliated Hospital of Anhui Medical University review board. The study reporting adheres to the laws of China. An informed consent form was signed by all participating patients regarding radiology, biopsies, cytology, and publication of the study, during hospitalization.

### Study population

**Figure 1:**
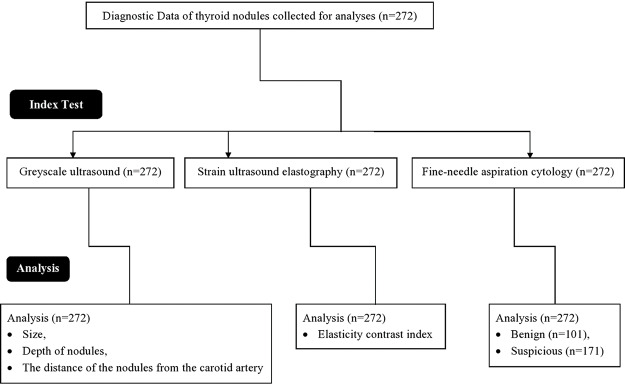
Flow diagram of the collection of data for the analysis.

From 15 August 2018 to 14 September 2019, a total of 234 patients (197 females and 37 males) underwent grayscale ultrasound, strain ultrasound elastography, and fine-needle aspiration cytology at the First Affiliated Hospital of Anhui Medical University, Hefei, China. Data were collected for the current study from the institute after approval from the competing authorities (Figure 1). The enrolled patients’ demographic and clinical information is displayed in Table 1.

### Grayscale ultrasound

**Figure 2:**
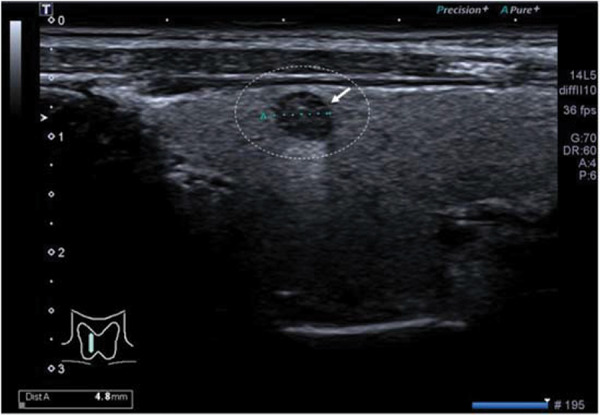
Grayscale ultrasound image of the right thyroid lobe of a 35-year-old female patient. The white circle and white arrow indicate a suspicious thyroid nodule. The maximum diameter of the nodule in the transverse view was 4.8 mm.

**Figure 3:**
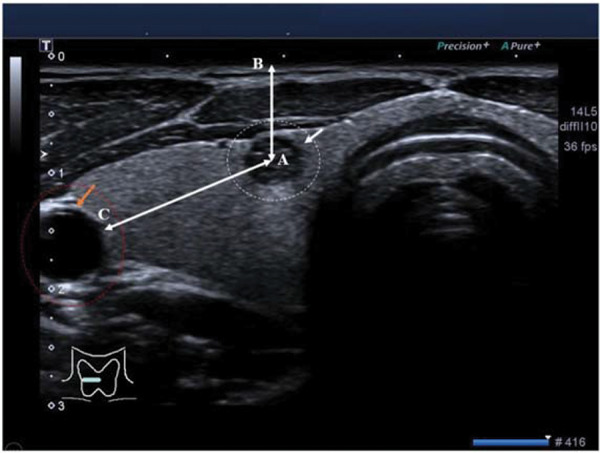
Grayscale ultrasound image of the right thyroid lobe of a 36-year-old female patient. The nodule was 3 mmin size. The white circle and white arrow indicate a suspicious thyroid nodule. The saffron circle and saffron arrow indicate the carotid artery. AB: the depth of the nodule and AC: the distance of the nodule from the carotid artery.

Grayscale ultrasound was performed by ultrasound technologists (a minimum of 3-years of experience) using Aplio 500 (Toshiba, Irvine, CA, USA) with an L5-14 MHz linear transducer. The size, depth, and the distance of the nodules from the carotid artery were evaluated. The maximum diameter of the nodules in the transverse view was the size of the nodules (Figure 2). The vertical distance from the center of the nodules to the skin was the depth of the nodules, and the ordinary straight-line distance from the center of the nodules to the carotid artery was the distance of the nodules from the carotid artery (Figure 3) (2).

### Strain ultrasound elastography

**Figure 4:**
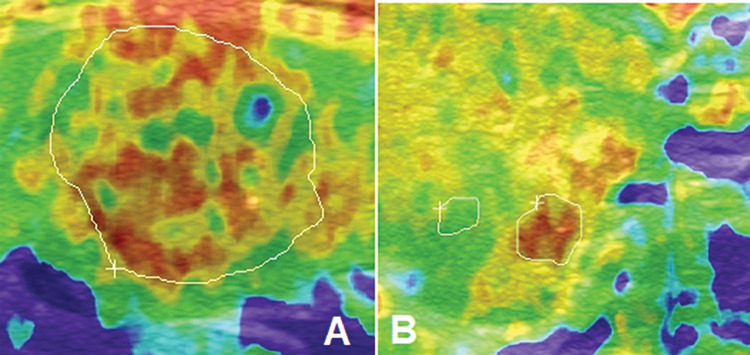
Strain ultrasound elastography of the thyroid gland. ASTERIA scoring criteria. **A**. Suspicious nodule located within 15 mm from the carotid artery (m=115). **B**. Suspicious nodule located more than 15 mm from the carotid artery (n=56).

Strain ultrasound elastography was performed by ultrasound technologists (a minimum of 3-years of experience) using Aplio 500 (Toshiba, Irvine, CA, USA). Using grayscale ultrasound, the transverse plane was identified, including the common carotid artery and thyroid gland. Patients were instructed to hold their breath, and the evaluator recorded data for 4s. The elasticity contrast index (the difference of tissue strain determined by elasticity imaging) was evaluated at the size of the nodule (Figure 4) (2). Scoring was given according to ASTERIA criteria as 1: Homogenously green (elasticity in the whole area), 2: The examined area was light green and red with a peripheral and central blue mass (the elasticity in the large portion of the area), 3: The examined area was blue with some light green and red mass (the large portion of the nodule with stiffness), 4: Homogeneously blue color (non-elastic nodule) (18).

### Fine-needle aspiration cytology

Under ultrasound guidance, fine-needle aspiration biopsies were performed by physicians (a minimum of 3-years of experience), and the biopsied material was sent for cytology. Cytologists (a minimum of 3-years of experience) performed the pathological analysis of the samples as per the 2017 Bethesda system for reporting thyroid cytopathology (19).

### Statistical analysis

SPSS, version 25 (IBM Incorporation, New York, USA) was used for the statistical analysis. A univariate regression analysis was performed between the nodule parameters and values of the elasticity contrast index. A multivariate regression analysis was performed between the nodule parameters and the diagnostic performance of the ultrasound elastography diagnosis (2). Continuous data were analyzed using a one-way analysis of variance (ANOVA). The Tukey test (considering critical value [*q*]>3.314 as significant) was used for the posthoc analysis. The results were considered significant at a 95% level of confidence.

## RESULTS

### Nodules parameters

A total of 272 nodules of 234 patients were screened by grayscale ultrasound and ultrasound elastography. Fine-needle aspiration biopsies found 101 benign nodules and 171 suspicious nodules. There was no significant difference in the depth of nodules (*p*=0.092) and the distance of the nodules from the carotid artery (*p*=0.061) between the benign and suspicious nodules. The suspicious nodules were smaller than the benign nodules (*p*<0.0001, *q*=23.84). The suspicious nodules had a higher elasticity contrast index than the benign nodules (*p*<0.0001, *q*=21.05). Ultrasound parameters of the benign and suspicious nodules are presented in Table 2.

### Nodule parameters and diagnostic performance of ultrasound elastography

The depth and the size of the nodules were not associated with the correct value of the elasticity contrast index (*p*>0.05 for both). When suspicious nodules were closed to the carotid artery, the elasticity contrast index was underestimated (*p*=0.009, Table 3). Therefore, all nodules were categorized as being less than or more than 15 mm from the carotid artery (30 benign nodules and 115 suspicious nodules, and 71 benign nodules and 56 suspicious nodules, respectively). The diagnostic performance of ultrasound elastography was not affected by the distance of the nodules from the carotid artery if the nodule was located 15 mm or more from the carotid artery (*p*=0.5960). However, if the suspicious nodule was located within 15 mm of the carotid artery, the diagnostic accuracy was hampered (*p*=0.006, Table 4).

**Figure 5:**
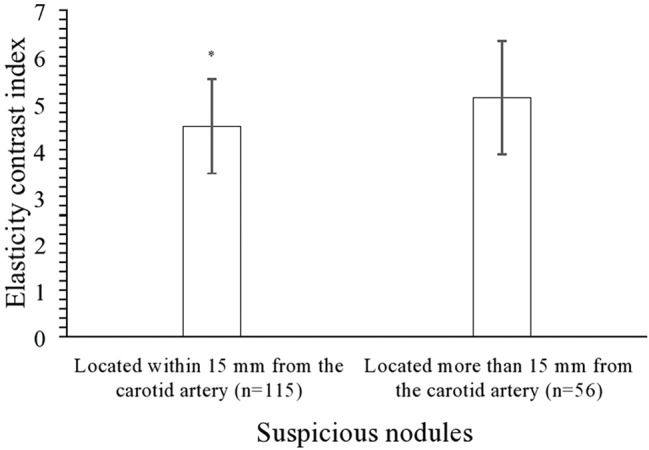
Comparative evaluation of the elasticity contrast index of suspicious nodules in relation to the distance from the carotid artery. Data are presented as a mean±SD. *Significantly lower.

The elasticity contrast index of the suspicious nodules located within 15 mm of the carotid artery was lower than that of suspicious nodules located 15 mm or more from the carotid artery (4.51±1.01 *versus* 5.12±1.21, *p*=0.0007, Figure 5).

## DISCUSSION

This study evaluated the effect of the size, depth, and distance of the nodules from the carotid artery (evaluated by grayscale ultrasound) on the performance of strain ultrasound elastography. We found that the distance of the nodules from the carotid artery was associated with the performance of strain ultrasound elastography. The results of the study were consistent with retrospective studies on thyroid nodules (2,3). The amount of force applied to the thyroid nodule depends on its distance from the compression source (2). Ultrasound elastography should be evaluated carefully when thyroid nodules are located near the carotid artery.

The study reported that the depth of the nodules did not affect the accuracy of the ultrasound findings. The results of the study were consistent with a retrospective study on thyroid nodules (2) and a prospective study on breast nodules (20). The depth of the nodules did not affect the management of the thyroid nodules.

The benign nodules were larger than the suspicious nodules. The results of the study were not consistent with some retrospective (2) and prospective (16) studies on thyroid nodules but were consistent with other retrospective studies (1,4,5) on thyroid nodules. Differences in the experimental setup and patient populations can explain these contradictory results. The malignancy factor is not directly correlated to the size of the nodules.

Suspicious nodules have a higher elasticity contrast index than benign nodules. The results of the study were consistent with retrospective (2,3,21,22) and prospective (23) studies on thyroid nodules. The elasticity contrast index is successful in the differentiation of benign and thyroid nodules.

The study also reported that the size of the nodule did not affect the accuracy of the ultrasound findings. The results of the study were consistent with retrospective (1,2,4,5) and prospective (16,20) studies on thyroid nodules. Ultrasound elastography could be used in the management of thyroid nodules.

In this study, nodules were categorized by their distance from the carotid artery (less than or more than 15 mm). In the diastole of the heart, the thyroid is contracted and expands in an axial direction. The natural pulsation of the carotid artery is a force source, and it attenuates with distance. This effect is observed in the thyroid nodule within 15 mm from the carotid artery (24). Therefore, this study used 15 mm from the carotid artery as the threshold.

This study has several limitations. As this is a retrospective analysis, there may be a chance of bias, and a dynamic study is required to confirm the findings. The nodules' size was measured on the transverse view only. The demographical and clinical conditions of the patients (2) and autoimmune thyroiditis (23) have been found to affect the diagnostic accuracy of ultrasound elastography. However, these parameters were not considered in this study. Intra-observer and interobserver agreements are required in ultrasound findings (3), but this study did not evaluate these parameters. The study did not consider the characteristics of the nodules, which can influence the elasticity, such as calcification, fibrosis.

## CONCLUSIONS

The depth and size of the nodules were not associated with the accuracy of the ultrasound findings. However, the distance of the nodules from the carotid artery significantly affected the accuracy of ultrasound elastography. Ultrasound elastography should be carefully evaluated when small thyroid nodules are located near the carotid artery (less than 15 mm).

## AUTHOR CONTRIBUTIONS

All authors read and approved the manuscript for publication. Yu Y was the project administrator and contributed to the conceptualization, methodology, resources, supervision, and literature review. Xie X contributed to the investigation, formal analysis, data curation, and literature review, and manuscript drafting, editing and review. Both authors agree to be accountable for all aspects of work, ensuring both integrity and accuracy.

## Figures and Tables

**Table 1:** Demographical parameters and clinical conditions of the enrolled patients.

Parameters		Value
Patients		234
Age (years)	Minimum	28
	Maximum	69
	Mean±SD	49.52±6.71
Gender	Male	37 (16)
	Female	197 (84)
Family history of thyroid nodule		17 (7)
Nodule per patient, average (range)		1.16 (1-4)

Categorical variables are shown as a frequency (percentage), and continuous variables are shown as a mean±SD.

**Table 2:** Grayscale ultrasound and strain ultrasound elastography parameters of the nodules.

Parameters		All nodules	Benign nodules	Suspicious nodule	Comparison between the benign and suspicious nodules	
					p-value	q-value
Nodules screened		272	101	171		
Size (mm)	Minimum	3.32	7.58	3.32	<0.0001	23.84
	Maximum	19.58	19.58	6.55		
	Mean±SD	3.91±1.12	5.55±1.23	3.28±0.88		
Depth of the nodule (mm)	Minimum	4.12	4.12	5.01	0.092	N/A
	Maximum	25.12	25.12	24.18		
	Mean±SD	9.27±1.28	09.15±1.68	9.48±1.01		
Distance of the nodules from the carotid artery (mm)	Minimum	6.12	8.15	6.12	0.061	N/A
	Maximum	28.22	28.22	25.45		
	Mean±SD	12.13±1.35	12.45±1.11	11.99±2.01		
Elasticity contrast index	Minimum	1.55	1.55	3.01	<0.0001	21.05
	Maximum	8.11	3.55	8.11		
	Mean±SD	3.61±1.18	2.55±0.95	4.75±1.29		

Variables are shown as a mean±SD.

A one-way analysis of variance (ANOVA) following the Tukey test was used for the statistical analysis.

A *p-*value of <0.05 and *q*>3.314 were considered significant.

N/A: Not applicable.

**Table 3:** The effect of the nodule parameters on the elasticity contrast index.

Type of nodules	All nodules		Benign nodules		Suspicious nodule	
Parameters	Coefficient	*p*-value	Coefficient	*p*-value	Coefficient	*p*-value
Nodules screened	272		101		171	
Size (mm)	0.059	0.452	0.051	0.623	0.061	0.561
Depth of the nodule (mm)	0.022	0.856	0.032	0.912	0.042	0.563
Distance of the nodules from the carotid artery (mm)	0.311	0.012*	0.123	0.062	0.412	0.009*

Univariate regression analysis.

A *p*-value of <0.05 was considered significant.

* Significant difference.

**Table 4:** Association of the nodule parameters on the ultrasound elastography diagnosis.

Type of nodules		All nodules			Benign nodules			Suspicious nodule		
Parameters		Odds ratio	95% CI	*p*-value	Odds ratio	95% CI	*p*-value	Odds ratio	95% CI	*p*-value
Nodules screened		272			101			171		
Size (mm)		0.923	0.822-0.985	0.652	0.895	0.812-0.923	0.589	0.623	0.544-0.824	0.625
Depth of the nodule (mm)		0.852	0.765-0.912	0.758	0.877	0.856-0.945	0.422	0.721	0.652-0.655	0.725
Distance of the nodules from the carotid artery (mm)	<15	1.811	1.556-2.125	0.038*	1.024	0.986-1.082	0.065	2.111	1.851-2.35	0.006*
	≥15	1.012	0.985-1.234	0.096	1.023	0.985-1.112	0.121	1.152	0.101-1.345	0.071

Multivariate regression analysis.

Cl: Confidence interval.

Odds ratio >1 and a *p*-value<0.05 were considered significant.

* Significant difference.
